# Novel Schiff base cobalt(ii) phthalocyanine with appliance of MWCNTs on GCE: enhanced electrocatalytic activity behaviour of α-amino acids†

**DOI:** 10.1039/d1ra01815a

**Published:** 2021-05-06

**Authors:** Mounesh, T. M. Sharan Kumar, N. Y. Praveen Kumar, K. R. Venugopala Reddy, K. B. Chandrakala, L. Arunkumar, C. C. Vidyasagar

**Affiliations:** Department of Studies and Research in Chemistry, Vijayanagara Srikrishnadevaraya University Ballari-583105 Karnataka India mounesh.m.nayak@gmail.com +91-8197546693; Department of Studies and Research in Chemistry, Ballari Institute of Technology and Management Ballari-583104 Karnataka India; Department of Studies and Research in Chemistry, Rani Channamma University Belagavi-591156 Karnataka India

## Abstract

A novel tetra-4-{(*E*)-[(8-aminonaphthalen-1-yl)imino]methyl}-2-methoxyphenol Co(ii) phthalocyanine (CoTANImMMPPc) was synthesized using a precursor protocol and characterized *via* electroanalytical and spectroscopic techniques. The FT-IR spectra of the synthesized compounds showed significant peaks corresponding to the functional groups of the precursors and phthalocyanine (Pc) compound. The mass and NMR spectra confirmed the formation of the target precursor compounds. A film of CoTANImMMPPc was deposited on the surface of an electrode and applied for the detection and monitoring of l-alanine and l-arginine. The cyclic voltammetric studies of l-alanine and l-arginine using the (CoTANImMMPPc/MWCNTs/GC) electrode showed a linear response in the range of 50–500 nM and the limit of detection was found to be 1.5 and 1.2 nM, respectively. Differential pulse voltammetry and chronoamperometry showed that the catalytic response for l-alanine and l-arginine is in the range of 50–500 nM with an LoD of 1.8 and 2.3 nM, respectively. The oxidation-active CoTANImMMPPc film significantly enhanced the current response in the chronoamperometric method and displayed a selective and sensitive response towards l-alanine and l-arginine in the presence of various other bio-molecules. The developed electrode showed good working stability and was applied for the analysis of real samples, which yielded satisfactory results. Therefore, CoTANImMMPPc-MWCNTs/GCE shows good analytical performance, is economical and produced *via* a simple synthetic method and can be applied as a sensor for the detection of l-alanine and l-arginine.

## Abstract 2

A novel CoTANImMMPPc complex was synthesized using a precursor protocol and characterized *via* electroanalytical and spectroscopic techniques with enhanced electrocatalytic behaviour of α-amino acids.

## Graphical abstract

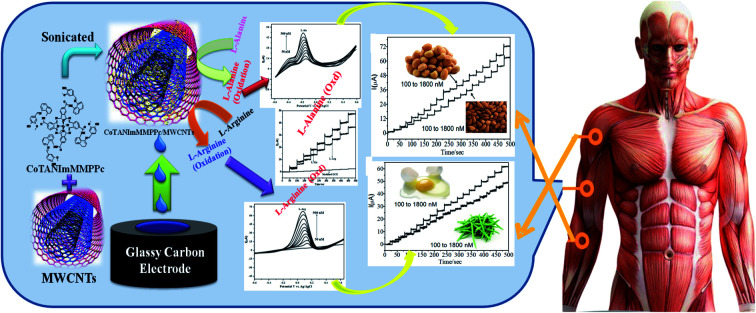

## 1. Introduction

Amino acids are an important class of organic compounds, which are required in numerous processes in biological systems and used as medicine and in food and beverages. Among them, alanine and arginine play very important roles in many biological functions. l-Alanine (l-Ala) is an amino acid used in the conversion of tryptophan and vitamin B6 into proteins, which provide energy for the muscles and increase immunity in the human body. As another amino acid, l-arginine (l-Arg) is transformed in the body into nitric acid, which acts as a neurotransmitter and makes the circulation of blood very easy by relaxing the blood vessels. Thus, considering the abovementioned importance of alanine and arginine, it is essential to study their electrochemical behavior and sensitive detection. Electrochemical investigations employing phthalocyanines have been reported by researchers, leading to an enhancement in the electrochemical process by changing their functional groups and forming composites with carbon materials such as graphite and carbon nanotubes.

The electrochemical oxidation of l-Ala and l-Arg on substituted phthalocyanines has been reported earlier.^1–6^ However, the electrochemical sensing is not satisfactory due to the slow movement of electrons at the electrode interface.^7–13^ Accordingly, it has been reported that the efficiency of phthalocyanines can be enhanced by forming composites with carbon particles such as graphite and carbon nanotubes. The modified electrodes can be used for oxidation and the determination of the adsorption behavior of amino acids. Many researchers have focused on the development of the electrocatalytic process, mainly enhancing the overpotential and faradaic current encountered in the electrooxidation of molecules using macrocyclic transition metal complexes.^14–18^

A cobalt tetracarboxylic acid phthalocyanine (CoTCAPc) was immobilized on a gold electrode for the oxidative and reductive detection of H_2_O_2_ at the physiological pH. We also reported the fabrication of glucose oxidase (GOx) enzyme on a gold electrode modified with an electrocatalyst CoTCAPc,^19^ cobalt phthalocyanine tetracarboxylic acid (CoPc-COOH) by H_2_O_2_,^20^ tetra chlorobenzoxazolamine nickel(ii) phthalocyanine (NiTCBPc),^21^ novel *n*-octadecylcarboxamide CoPc for the amperometric detection of bioanalytes using a modified GCE (CoODAPc),^22^ cobalt(ii) tetrasulfanilamide phthalocyanine,^23^ cobalt phthalocyanine tetracarboxylic acid-functionalized polymer monolith for the selective enrichment of glycopeptides and glycans,^24^ and the importance of electrochemical methods in biological and environmental analyses for the determination biomolecules.^25–27^

In this work, we focused on the synthesis of a CoTCAPc complex substituted with the Schiff ligand 4-{*E*-[(8-aminonaphthalen-1-yl)imino]methyl}-2-methoxyphenol (ANImMMP). The structure of the compound was confirmed *via* FTIR, UV-visible, XRD, TGA, mass spectroscopy, and elemental analysis. The Pc complex was used to form a composite with MWCNTs and employed for the detection of nanomolar concentrations of l-Ala and l-Arg *via* the cyclic voltammetry (CV), differential pulse voltammetry (DPV) and chronoamperometry (CA) techniques. Specifically, the modified CoTANImMMPPc/GCE with multiwalled carbon nanotubes (MWCNTs/CNTs) was employed for the detection of nanomolar concentrations of l-Ala and l-Arg *via* the CVs, DPV and CA techniques. The selectivity studies in the presence of some biomolecules including ascorbic acid, dopamine, l-cysteine, l-asparagine (l-Asp), glucose and hydrogen peroxide showed negligible current responses by l-Ala and l-Arg at nM concentrations. In the present work, the oxidation of the l-Ala and l-Arg analytes exhibited well separated and defined peaks. Also, we focused on the surface modification technique in the electrochemical system, which is significant for experimental design, to build an electrochemical sensor with high selectivity, low detection limit, excellent linear concentration responses, reproducibility and sensitivity for the simultaneous detection of individual analytes and l-Ala in the presence of l-Arg.

## 2. Experimental

### 2.1 Materials

1,8-Diaminonaphthalene, *m*-vanillin, methanol, l-Ala, l-Arg, l-asparagine (l-Asp), l-cysteine (l-Cys), glucose (GOx), hydrogen peroxide (H_2_O_2_), ascorbic acid (AA), dopamine (DA), anhydrous potassium carbonate (K_2_CO_3_), hexane, tetrahydrofuran (THF) and *N*,*N*′-dicyclohexylcarbodiimide (DCC), dimethyl sulfoxide (DMSO) were supplied by Sigma Aldrich. *N*,*N*′-Dimethylformamide (DMF) was obtained from M-Tedia (USA) and used without further purification.

### 2.2 Preparation of ANImMMP and CoTANImMMPPc

**Scheme 1:**
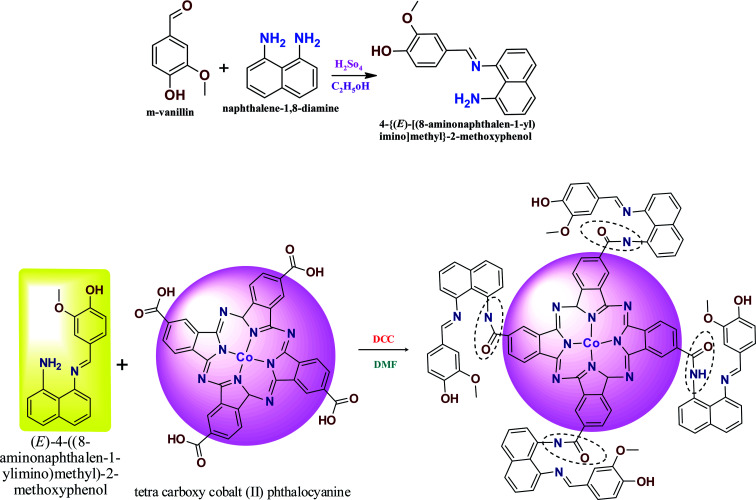
Preparation of ANImMMP and CoTANImMMPPc.

The novel Schiff base ligand was synthesized by adding *m*-vanillin (1 g, 0.0055 M), diaminonaphthalene (1.05 g, 0.0055 M) and 20 mL of methanol to a round-bottom flask. The mixture was stirred under a nitrogen atmosphere, and subsequently 1–2 mL of H_2_SO_4_ was added dropwise. The chemical mixture was stirred for 6 h at 45–50 °C to obtain a bright gray precipitate. The chemical mixture was refluxed under vacuum and washed with water and recrystallized by methanol and purified by column chromatography using hexane and ethyl acetate as the solvent. Yield (1.298 g, 78.1%). Melting point: 197–200 °C of ANImMMP (Scheme 1).^28,29^

The CoTANImMMPPc complex was synthesized by adding CoTCAPc (0.52 g, 0.00069 M), K_2_CO_3_ (0.48 g, 0.00345 M) and DCC catalyst dissolved in DMF (25 mL) to an RB flask. The reaction mixture was stirred for 25 min, then ANImMMP (1.5 g, 0.00343 M) was added to the reaction mixture and the solution was stirred for 46 h at 28 °C. A dark green precipitate was formed, and then the product was filtered and washed with cold water and hot water followed by hexane to give CoTANImMMPPc in a yield of 95% (Scheme 1).^1,30^

### 2.3 Characterization methods

A Shimadzu UV-2550 spectrophotometer is used to measure the UV-visible absorption spectra and the FTIR spectra were measured using a PerkinElmer spectrum 100 FTIR spectrometer. ^1^H-NMR spectra were recorded at 300 MHz on a Bruker spectrometer and the chemical shift values are expressed in *δ* ppm with respect to TMS as an internal standard. X-ray diffraction (XRD) (CuKα radiation) patterns were measured using a Bruker Advanced D8-diffractometer. The mass spectrum of the synthesized compound was confirmed using an ESI-MS MALDI-Micro mass Q-TOF2 instrument. Thermogravimetric analyses (TGA) were performed on a Mettler-Toledo instrument at a heating rate of 25 °C min^−1^ and nitrogen flow rate of 40 mL min^−1^. All electrochemical analyses were performed on a CHI620E electrochemical workstation USA with a conventional 3-electrode system (glassy carbon electrode (GCE), Ag/AgCl electrode and platinum electrode).

### 2.4 GCE surface modification technique

The surface of the GCE was rubbed with a 0.6 mm alumina slurry and then completely washed with distilled water and sonicated in water followed by acetone for about 5 min followed by drying in an oven at 25 °C. Then 5 mg of CoTANImMMPPc and Nafion binder were ultrasonicated for 30 min for their dispersion in DMF. Using the drop-coating method, the CoTANImMMPPc material was deposited on the GCE electrode. Then the electrode was dried at 25 °C. The same procedure used for the preparation of the CoTANImMMPPc-MWCNT electrode using MWCNTs/CNTs. These modified electrodes were used for the electrochemical detection of l-Ala and l-Arg.

## 3. Results and discussion

The preparation of the ANImMMP and CoTANImMMPPc complex is presented in Scheme 1. Specifically, vanillin reacts with diaminonaphthalene to form an imine bond. The ligand and complex were obtained with high purity and good yield and were characterized using different spectral techniques including FT-IR, ^1^H NMR, TGA, MASS, P-XRD and UV-visible spectroscopy. The carboxylic group of the CoTCAPc amine group reacted with the ligand to produce the amide-bridged CoTANImMMPPc complex. The elemental analysis of the synthesized complex gives evidence for the purity of the complex and the experimental values are consistent with the theoretical values, as shown in Table S1.† The CoTANImMMPPc complex is dark green in color and completely soluble in concentrated sulfuric acid (H_2_SO_4_) and DMSO.

### 3.1 FT-IR spectra

The FT-IR (cm^−1^) spectrum in Fig. S1a† shows intense and broad absorption bands in the region of 3680–3400 cm^−1^ (–OH and –NH_2_). In the spectrum shown in Fig. S1b,† the peak corresponding to the carboxylic acid group of CoTCAPc appears in the range of 3700–3200 cm^−1^. However, in Fig. S1c,† the peak for the –COOH group disappears with the appearance of a peak corresponding to a substituted amide group (CoTANImMMPPc) at 3327 cm^−1^ (–CONH), a peak in the region of 2934–2663 cm^−1^ (Ar-CH), and peaks for the vibrations caused by the stretching of the (C=N) and (C=C) at around 1631–1606 cm^−1^. The sharp peak in the region of 1565–1523 cm^−1^ corresponds to C=O, and the sharp peak at 744 cm^−1^ is attributed to C–Br. Thus, the vibrational bands at 1499, 1457, 1433, 1392, 1309, 1245, 1228, 1113, 1032, 884, 847, 844, 647, and 605 cm^−1^ support the presence of functional groups in the CoTANImMMPPc ring.

### 3.2 ^1^H NMR spectra

^1^H-NMR (300 MHz, DMSO-d_6_): *δ* 2.50 (3H, s), 6.68 (1H, dd, *J* = 7.8, 1.6 Hz), 6.80 (1H, dd, *J* = 8.4, 0.5 Hz), 6.91 (1H, dd, *J* = 7.8, 1.3 Hz), 6.99 (1H, dd, *J* = 1.7, 0.5 Hz), 7.20 (5H, 7.48 dd, *J* = 8.4,1.7 Hz), 7.30 (td, *J* = 7.8, 0.5 Hz), 7.96 (ddd, *J* = 8.1, 7.8, 0.5 Hz), 8.12 (dddd, *J* = 8.1,2.0,1.3, 0.5 Hz), 8.43 (1H, s), 4.10 (1H, s) and 2.0 (base line), as shown in Fig. S2.†

### 3.3 UV-visible spectra

The UV-Vis spectra of the ANImMMP, CoTCAPc and CoTANImMMPPc systems show distinct B and Q bands. ANImMMP shows the Q-band at 300–500 nm and B-band at 200–260 nm (Fig. S3† inset a curve). The UV studies of the phthalocyanine exhibit two strong absorption curves, where one appears in the range of 550–720 nm (Q band), which represents the π–π* transition from the HOMO to the LUMO within the Pc ring. The second curve in the wavelength range of 300–450 nm corresponds to the B band (Fig. S3,† inset curves b and c), arising from the deeper π-levels/LUMO transition.^31,32^ The UV-Vis spectrum of the compound shown in Scheme 1 in DMSO at 28 °C is presented in Fig. S3.† The red and green color of the complexes show a peak in the Q-band region and a shoulder peak was observed in the range of 550–700 nm, indicating the good aggregation of Pcs.

### 3.4 PXRD analysis

The powder X-ray diffraction study (PXRD) of CoTANImMMPPc was done in the 2*θ* range of 10–100°, as shown in Fig. S4,† inset curve ((a) ANImMMP, (b) CoTCAPc and (c) CoTANImMMPPc). The PXRD analysis was performed to elucidate the crystal nature and size of the QDs. The parent Pcs and substituted complex exhibit the same patterns. However, the patterns vary in intensity for the complex compared to the corresponding metal Pcs. The PXRD patterns are used to describe the crystallinity of materials.^33,34^ The diffraction pattern of CoTANImMMPPc shows sharp peaks at 9°, 20°, 21°, 25°, 49°, 60°, and 70° with a low intensity, indicating that CoTANImMMPPc is crystalline in nature. Furthermore, the shapes of the X-ray diffraction patterns indicate that ANImMMP, CoTCAPc and CoTANImMMPPc were crystalline in nature.

### 3.5 Thermogravimetric analysis

Fig. S5† shows the thermal stability and decomposition behavior of the CoTANImMMPPc and CoTCAPc complexes at various temperatures (inset (a) CoTCAPc and (b) CoTANImMMPPc). The TGA data shows that CoTANImMMPPc and CoTCAPc degraded mainly in three ways in a nitrogen environment. The first step revealed that the initial weight loss of 0% corresponds to the moisture of volatile species. In the second step, the substituent gets isolated in the temperature range of 0–386 °C, leading to 55.14% weight loss due to the degradation of the substituted ligand. The third step occurs readily in the oxidizing environment and leads to the degradation of the Pc structure in the temperature range of 386–580 °C and 24.46% weight loss. Finally, the cobalt oxide (CoO) product is formed, and the cobalt oxide corresponds to 21.42% weight loss. Thus, all these results show that the substituted CoTANImMMPPc has greater stability compared to other substituted metal phthalocyanines.^35–38^

### 3.6 Mass spectra

LC-mass spectrum (LCMS) analysis: *m*/*z* [M] calcd. 292 for C_18_H_16_N_2_O_2_: found [M + Z] +293, as shown in Fig. S6† for ANImMMP. The mass spectrum of CoTANImMMPPc shown in Scheme 1 confirms the desired structure: *m*/*z* [M] calcd. 1851 for C_108_H_79_CoN_16_O_12_: found [M + Z] +1853, as shown in Fig. S7.†

## 4. Electrochemical studies

**Fig. 1:**
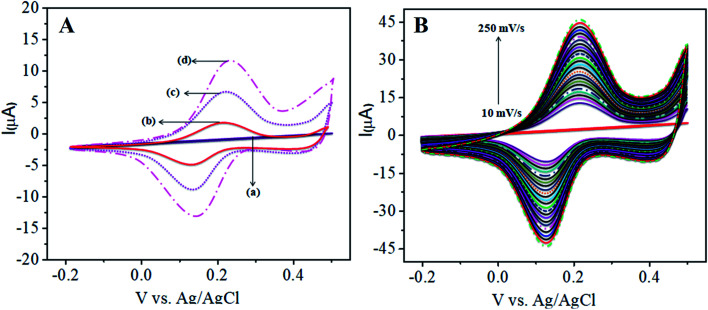
Cyclic voltammetric curves in (pH 7) PBS for 100 nM K_4_Fe(CN)_6_: (A) inset curves (a) bare GCE without K_4_Fe(CN)_6_ in (pH 7) PBS, (b) bare GCE, (c) CoTANImMMPPc/GCE, (d) CoTANImMMPPc/CNTs/GCE at a scan rate of 50 mV s^−1^ and (B) various scan rates for CoTANImMMPPc/CNTs/GCE/mV s^−1^.

Fig. 1A shows the charge transfer behavior of the CoTANImMMPPc/GCE and CoTANImMMPPc-CNTs/GCE electrodes, where in the CVs plot no peak can be observed at the bare GCE in pH 7 PBS solution (inset curve a). Conversely, when the same reaction was carried out in the presence of the K_4_Fe(CN)_6_ system in 100 nM, a peak corresponding to the [Fe(CN)_6_^3−^]/[Fe(CN)_6_^4−^] redox was observed (Fig. 1A, inset curve b). Then the GCE surface was well-coated with CoTANImMMPPc and CoTANImMMPPc-CNTs, and the modified GCE electrodes were immersed in PBS containing 100 nM K_4_Fe(CN)_6_ (Fig. 1A, inset curve c). The modified GCE and with CNTs exhibited the fast movement of electrons in the [Fe(CN)_6_^3−^]/[Fe(CN)_6_^4−^] redox couple system, which was not inhibited by the CoTANImMMPPc/CNTs. The lack of inhibition of the redox couple was observed even before the substituted CoPc was deposited on the GCE; however, both CoTANImMMPPc/GCE and CoTANImMMPPc/CNTs/GCE show high peak current intensities and the same redox potential for the [Fe(CN)_6_^3−^]/[Fe(CN)_6_^4−^] system at the scan rate of 50 mV s^−1^ because the modified GCE acts as a current carrying conductor and it allows rapid electron transfer in solution. The modified CoTANImMMPPc-CNTs/GCE was scanned at various scan rates, and a linear increase in the peak current was observed in both the anodic and cathodic peak currents, showing a positive potential at 240 and 130 mV with an increase in the scan rate (10–250 mV s^−1^), respectively, as shown in Fig. 1B. Thus, the increase in peak current observed in the CV plot with the square root of scan rate indicates a diffusion-controlled mass transfer process.^39,40^

### 4.1 Electrochemical characterization

The electrochemical investigation of the CoTANImMMPPc-modified electrode was carried out in the presence of (pH 7) PBS, bare GCE (inset Fig. S8A†). CoTANImMMPPc/GCE exhibited a cathodic peak potential of 25 mV with low current responses (Fig. S8A,† inset curve a), and CoTANImMMPPc/CNTs/GCE exhibited an enhanced peak current, as shown by the cathodic peak potential (Fig. S8A,† inset curve b) at the scan rate of 50 mV s^−1^. The electrons transfer to the CNTs absorbed on the hydrophobic surface of the GCE by Co^III^/Co^II^. On the CoPc–CNTs-modified GCE, the oxidation of CoTANImMMPPc-CNTs/GCE in pH 7 PBS occurs in the one-step electrocatalytic oxidation of Co(ii)Pc to Co(iii)Pc according to eqn (1) as follows:

1 Co^II^PC → Co^III^PC + e^−^

The oxidation of the CoTANImMMPPc/CNT electrode was observed using the cathodic peak potential by applying different scan rates in the range of 10–100 mV s^−1^ with an increase in the high positive current response by CoTANImMMPPc/CNTs. The linear regression curve determined using *I*_p_*vs.* different scan rates was *Y* = 0.224*x* + 25.610 with a correlation coefficient of *R*^2^ = 0.998 (inset Fig. S8B†).

### 4.2 Nanomolar detection of l-alanine (l-Ala)

**Fig. 2:**
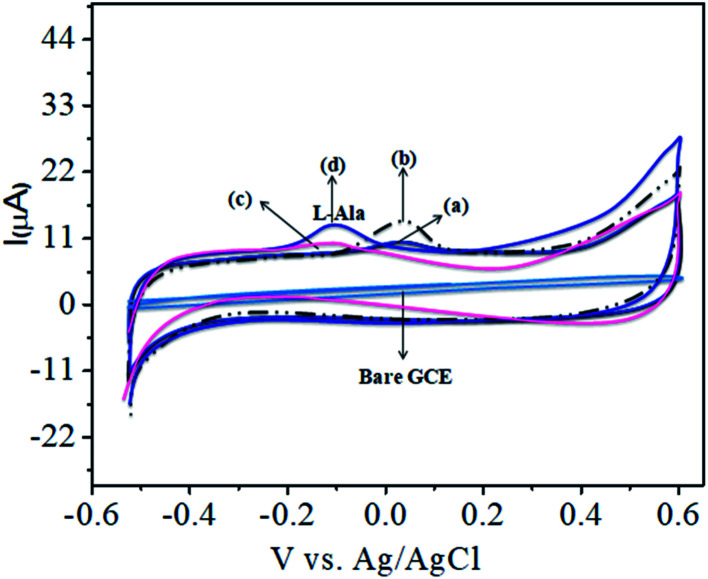
Experimental CVs of modified GCE in (pH 7) PBS at peaks: inset bare GCE, (a) CoTANImMMPPc, (b) CoTANImMMPPc/CNTs, (c) 100 nM of l-Ala by CoTANImMMPPc and (d) l-Ala by CoTANImMMPPc/CNTs at a scan rate of 50 mV s^−1^.

The CoTANImMMPPc complex was used for analytical applications, where both CoTANImMMPPc/GCE and CoTANImMMPPc/CNTs/GCE showed high peak current intensities due to the fast electron transfer, as discussed above (Fig. S8A and S2A,† inset curves a and b). The electrocatalytic ability of CoTANImMMPPc/CNTs was evaluated for the electrooxidation of l-Ala. Fig. 2 shows the cyclic voltammograms of CoTANImMMPPc (inset c curve) and CoTANImMMPPc/CNTs (inset d curve) in the presence of 100 nM l-Ala, where a strong oxidation peak was observed at −120 mV due to the high positive peak current of CoTANImMMPPc/CNTs and an increase in oxidation peak current was achieved for the oxidation of l-Ala. The well-defined anodic peak potential at −120 mV shows its significant electrocatalytic effect and good electrochemical response for detection of l-Ala.^41^ Thus, according to the results, in the presence of CNTs, a greater enhancement in peak potential was observed compared with CoTANImMMPPc.

**Fig. 3:**
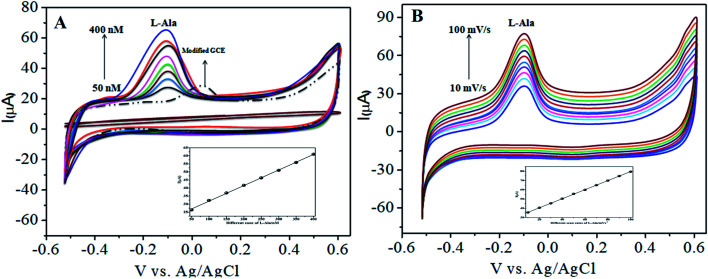
Experimental CVs of modified GCE in (pH 7) PBS electrolyte: (A) inset modified CoTANImMMPPc, various concentrations of 50–400 nM of l-Ala by CoTANImMMPPc/CNTs at scan rate 50 mV s^−1^ and (B) various scan rates (10–100 mV s^−1^) for the detection of l-Ala by CoTANImMMPPc/CNT electrode.

In our present work, the CoTANImMMPPc/CNT complex exhibits excellent electrocatalytic activity and it can facilitate the transfer of electrons in l-Ala, where the CoTANImMMPPc/GCE surface modulates the electrochemical reactions in a controlled fashion. In addition, the high density and well distributed CoTANImMMPPc on the surface of the CNTs can induce the exposure of more active sites for the catalytic oxidation reaction and result in efficient electrical behavior through direct binding with the CNTs, which enhances the electrocatalytic activity. Overall, the results show that both CNTs and CoTANImMMPPc play an important role and exhibit a synergistic effect in the oxidation of l-Ala in PBS (pH 7) solution. Fig. 3A shows the detection of l-Ala with different concentrations in the range of 50–400 nM at the anodic peak potential (−120 mV) with a high positive current, which indicates the excellent electrocatalytic oxidation of l-Ala by the modified GCE. The linear concentration range was determined to be 50–400 nmol L^−1^ using *I*_p_*vs.* different concentration: *Y* = 0.097 (l-Ala) + 22.144 with *R*^2^ = 0.999 (inset Fig. 3A) at different scan rates for the detection of 200 nM l-Ala at the anodic potential. With an enhancement in the scan rate (10–100 mV s^−1^) with a high positive peak current (Fig. 3B), the linearity was determined using *I*_p_*vs.* different scan rates: *Y* = 0.489*x* + 30.750 with the correlation coefficient of *R*^2^ = 0.9998 (inset Fig. 3B).^13^ Thus, the modified electrode exhibits a low detection limit and limit of quantification and high sensitivity, as shown in Table 1.

**Table 1:** Analytical parameters observed for the detection of l-alanine and l-arginine^a^

Method	Analyte	Detection methods	^a^Potential (*E*_p_)	^b^LOD	^c^LOQ	^d^ *R* ^2^	Sensitivity (μA nM^−1^ cm^−2^)	Linear range	Ref.
MWCNT-HF/QD modified PGE		DPV	−540	0.158 μM	0.610 μM	0.994	—	0.561–33 670 μM	43
Acid co-injection		Anion-exchange column	—	10.4 μM	—	0.999	—	0.5 to 20 μM	48
MWCNT-Cu_2_O CPE		CA	—	0.17 μM	—	—	—	5–400 μM	49
NiONPs/GCE		CA	0.42 V (AP)				0.4 nA μM^−1^ cm^−2^	30–200 μM	51
CoTANImMMPPc/CNTs/GCE		CVs	−0.120 V	1.5 nM	4.5 nM	0.999	0.097	50 to 400 nM	This work
		DPV	−0.120 V	1.8 nM	5.4 nM	0.997	0.081	50 to 500 nM	
		CA	−0.130 V (AP)	3.1 nM	9.3 nM	0.997	0.068	50 to 500 nM	
MWCNT-HF/QD modified PGE		DPV	−0.150 V	0.081 μM	0.312 μM	0.988	—	0.287–17 220 μM	43
Acid co-injection		Anion-exchange column	—	15.4 μM	—	0.992	—	0.5 to 8 μM	48
Iridium nano-CPE		CV, CA	—	19.7	—	—	—	0–544	50
CoTANImMMPPc/CNTs/GCE		CVs	+0.140 V	1.2 nM	3.6 nM	0.998	0.054	50 to 500 nM	This work
		DPV	+0.140 V	2.3 nM	6.9 nM	0.999	0.104	50 to 500 nM	
		CA	+0.150 V (AP)	3.5 nM	10.5 nM	0.997	0.105	50 to 500 nM	

a a = peak potential, b = limit of detection, c= limit of quantification, d = correlation coefficient, CV = cyclic voltammetry, DPV = differential pulse voltammetry, CA = chronoamperometry, AP = applied potential/fixed potential.

### 4.3 Detection of l-Ala in the presence of l-Arg

**Fig. 4:**
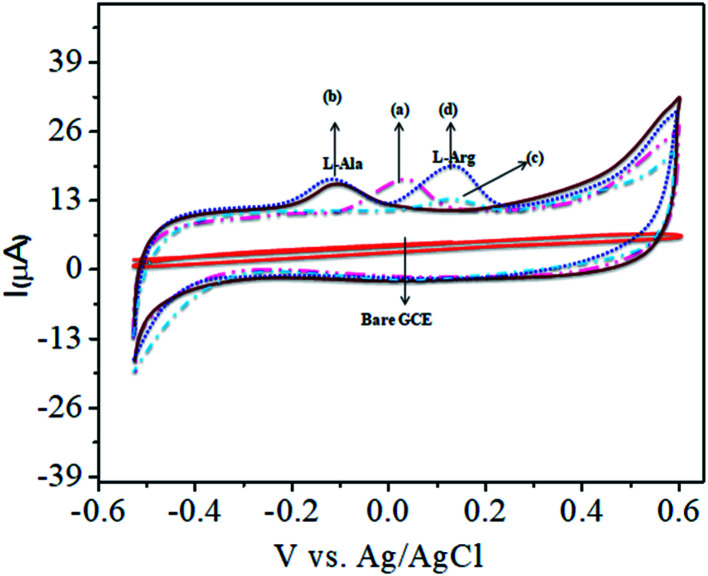
Experimental CVs of modified GCE in (pH 7) PBS: inset bare GCE, (a) CoTANImMMPPc/CNTs, (b) 100 nM of l-Ala by CoTANImMMPPc/CNT/GCE, (c) 100 nM of l-Arg by CoTANImMMPPc and (d) l-Ala in the presence of 100 nM of l-Arg by CoTANImMMPPc/CNTs at the scan rate of 50 mV s^−1^.

The analytical applicability of the CoTANImMMPPc/CNTs/GCE at +25 mV (Fig. 4, inset curve a) for the detection of l-Ala at −120 mV (Fig. 4, inset curve b) was investigated with the continuous addition of 100 nM l-Arg in the same electrolyte cell. The detected cathodic peak potential was +140 mV by CoTANImMMPPc (Fig. 4, inset curve c), whereas the CoTANImMMPPc/CNT electrode exhibited a high positive current at the oxidation peak potential compared to CNTs due to the good electrocatalytic activity of the material (Fig. 4, inset curve d). Initially, the electrochemistry for l-Ala and l-Arg on the surface of the modified GCE was studied *via* CV. A well-defined cathodic peak potential was observed at +140 mV with a remarkable increase in peak current due to the oxidation of l-Arg when the electrode surface was modified with the CoTANImMMPPc/CNT complex, which indicates its significant electrocatalytic effect and good electrochemical response for the oxidation of l-Arg in the presence of l-Ala.^42^

**Fig. 5:**
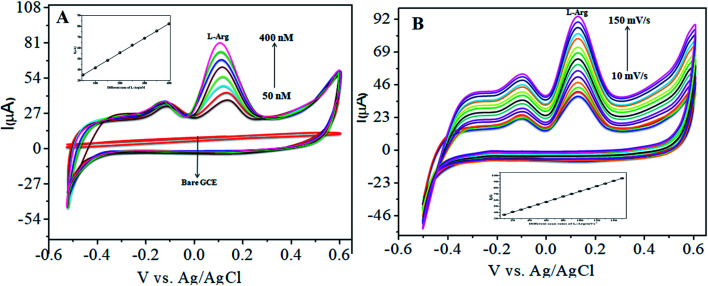
Experimental CVs of modified GCE in (pH-7) PBS: (A) inset bare GCE, l-Ala in the presence of various concentrations in the range of 50–400 nM of l-Arg by CoTANImMMPPc/CNTs at a scan rate of 50 mV s^−1^ and (B) various scan rates for the detection of l-Arg.

The CoTANImMMPPc/CNT electrode plays an important role and has a synergistic effect in the oxidation of l-Ala in presence of l-Arg in PBS (pH 7) solution. With a fixed concentration of l-Ala in the same cell and the addition of different concentrations in the range of 50–400 nM for the detection of l-Arg at the cathodic peak potential (+140 mV), the high positive current, as shown in Fig. 5A, indicates the excellent electrocatalytic oxidation of l-Ala simultaneously in the presence of l-Arg by the modified electrode, and the linear regression curve determined using *I*_p_*vs.* different concentration/nM was *Y* = 0.135 (l-Arg) + 28.432 with *R*^2^ = 0.99982 (inset Fig. 5A) at different scan rates for the detection of 200 nM l-Arg at the cathodic potential. With an enhancement in the scan rate (10–150 mV s^−1^) with a high positive current (Fig. 5B), the linearity *I*_p_*vs.* different scan rates was determined to be *Y* = 0.424*x* + 31.909 with *R*^2^ = 0.99986 (inset Fig. 5B). Thus, the CoTANImMMPPc-CNT electrode exhibits good electrocatalytic activity, reproducibility and stability.

#### 4.3.1 Individual detection of l-arginine

**Fig. 6:**
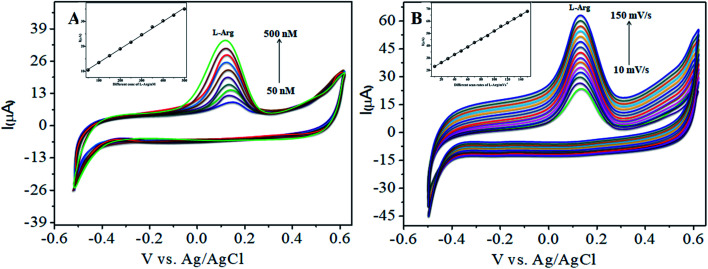
Experimental CVs of modified GCE in (pH 7) PBS: (A) inset bare GCE, various concentrations of l-Arg in the range of 50–500 nM by CoTANImMMPPc/CNTs at a scan rate of 50 mV s^−1^ and (B) various scan rates for the detection of l-Arg.

The electrocatalytic activity of CoTANImMMPPc/CNTs was examined *via* CV. Fig. 6 shows the CV responses for various concentrations of l-Arg in the range of 50–500 nM and CoTANImMMPPc/CNTs in (pH 7) PBS at a scan rate of 50 mV s^−1^ (Fig. 6A). The modified GCE electrode shows significant oxidation currents at +140 mV *vs.* Ag/AgCl and a reduction peak was not observed in the reverse scan. The substantial positive shift in the peak potential and enhancement in the current indicate the significant electrocatalytic ability of CoTANImMMPPc-CNTs for the oxidation of l-Arg, which can be attributed to the high surface area to volume ratio of the CoTANImMMPPc-CNT electrode, where l-Arg can easily penetrate the conductive porous channels of the electrode, leading to good sensitivity. The modified GCE was predicted to show a high cathodic peak current with an increase in the concentration of l-Arg in the range of 50–500 nM L^−1^ and the linearity *I*_p_*vs.* different concentrations of l-Arg was determined to be *Y* = 0.054 (l-Arg) + 8.008 with *R*^2^ = 0.998 (inset Fig. 6A). The CVs of 50 nM l-Arg solution at different scan rates (10–150 mV s^−1^) were recorded, as shown in Fig. 6B, and current function was smoothly enhanced with the potential sweep rate, confirming the electrocatalytic nature of the electrooxidation process, with the linear equation *Y* = 0.323*x* + 19.850 with *R*^2^ = 0.999. Thus, the modified GCE was exhibited good electrocatalytic activity and excellent analytical performance, as shown in Table 1.

### 4.4 DPV studies for l-Ala and l-Arg

**Fig. 7:**
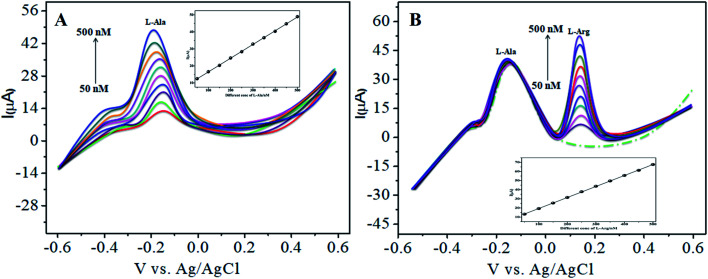
Experimental DPV for CoTANImMMPPc/CNT/GCE in (pH 7) PBS: (A) various concentrations of l-Ala in the range of 50–400 nM and (B) in the presence of different concentrations of l-Arg at a scan rate of 50 mV s^−1^.

The various parameters were studied in the DPV method by analyzing the peak currents and standard concentrations of the two analytes. For the simultaneous detection of l-Arg and l-Ala in PBS (pH 7) at a scan rate 50 mV s^−1^ in the presence of CoTANImMMPPc/CNTs, well-defined peaks were observed with a variation in the concentration, clearly indicating the effect of concentration. The simultaneous and individual voltammetric detection of l-Arg (Fig. 7A), and different concentrations of l-Ala (50–500 nM) using the CoTANImMMPPc/CNTs electrode was investigated at the anodic peak potential (−130 mV) by DPV, and the linear equation was determined to be *Y* = 0.081 (l-Ala) + 8.40 with *R*^2^ = 0.997 (inset Fig. 7A). When the concentration of one species changed, the concentration of l-Ala remained constant. Fig. 7B shows the various DPV of l-Ala with various concentrations in the presence of l-Arg analyte, where the peak currents for l-Arg increased linearly with an increase in l-Arg concentration in the range of 50–500 nM with the related regression equation *Y* = 0.120 (l-Arg) + 7.354 with *R*^2^ = 0.999, as shown in the inset of Fig. 7B.^43,44^

**Fig. 8:**
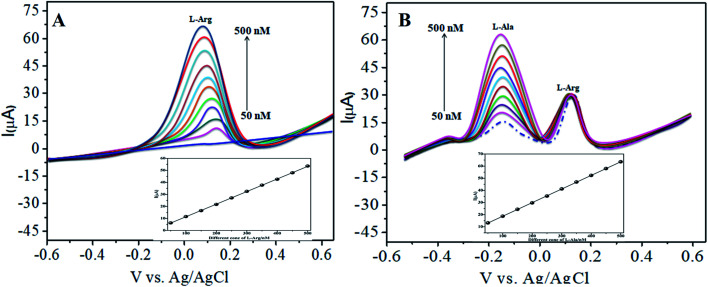
Experimental DPV of CoTANImMMPPc/CNTs in (pH 7) PBS: (A) various concentrations of l-Arg and (B) in the presence of different concentrations of l-Ala at a scan rate of 50 mV s^−1^.

Similarly, Fig. 8A and B show that the peak current was enhanced linearly with an increase in the concentration of l-Arg and l-Ala, and with a fixed concentration of l-Arg analyte constant. The results show that the electrochemical signals of l-Arg and l-Ala are not dependent on each other at the CoTANImMMPPc/CNTs electrode, where different concentrations (50–500 nM) of l-Arg, as shown in Fig. 8A, were detected at a constant cathodic peak potential (+140 mV), and as shown in Fig. 8B, l-Ala in the presence of a fixed concentration of l-Arg was detected at the anodic peak potential (−140 mV). Therefore, the selective determination of each amino acid in the presence of each other is possible and a well-distinguished anodic peak and cathodic peak corresponding to l-Arg and l-Ala oxidation can obtained at CoTANImMMPPc/CNTs, respectively. For the pre-concentration factor, the corresponding regression equation is *Y* = 0.104 (l-Arg) + 0.889, as shown in the inset of Fig. 8A, and *Y* = 0.112 (l-Ala) + 7.477, as shown in the inset of Fig. 8B with the correlation coefficient of 0.9998 and 0.999, respectively. Furthermore, the experimental limit of detection (LOD),^43,44^ quantification (LOQ), and linear dynamic range (LDR) were studied under the optimum conditions to evaluate the practical applicability of the sensor, as shown in Table 1.

### 4.5 Amperometric responses for l-Ala and l-Arg

**Fig. 9:**
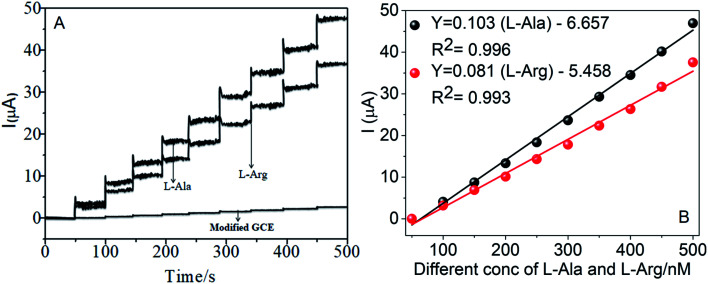
Individual amperometry responses of CoTANImMMPPc/CNT electrode in (pH 7) PBS: (A) inset modified GCE, various concentrations of l-Ala & l-Arg in the range of 50–500 nM and (B) linear plot of various concentrations of l-Ala and l-Arg/nM *vs.* peak current at the applied potential of ±150 mV.

The amperometric determination of l-Ala and l-Arg in flow systems and the hydrodynamic behavior of different concentrations in the range of 50–500 nM of l-Ala and l-Arg were investigated at the CoTANImMMPPc/CNT electrode, and the applied potential for l-Ala and l-Arg (±150 mV) is shown in the inset of Fig. 9A. For the detection of l-Arg, low current responses to l-Ala were observed, indicating that the oxidation of l-Ala readily increases at the modified GCE electrode due to electrocatalysis.^45,46^ Hence, a potential of −150 mV and +150 mV were selected as the working potential for the amperometry determination of l-Ala and l-Arg using CoTANImMMPPc/CNTs under hydrodynamic conditions, respectively. Fig. 9A shows the typical current–time responses of CoTANImMMPPc/CNTs during the successive addition of l-Ala and l-Arg separately to a continuous stirring PBS solution under the optimized experimental conditions (pH 7, applied potential of +150 and −150 mV *vs.* Ag|AgCl). It was observed that the sensor exhibited a response within 5 s (inset Fig. 9A). The sensor showed a linear response in the l-Ala and l-Arg concentration range of 50 to 500 nM L^−1^ with the linear equation *Y* = 0.068 (l-Ala) + 3.164 and *Y* = 0.105 (l-Arg) − 5.847 with a correlation coefficient of 0.997 and 0.997, respectively, as shown in Fig. 9B. Furthermore, the detection limit (signal/noise ratio [S/N] = 3) was found to be 120 and 100 nM L^−1^, respectively. Thus, the results indicate that our proposed sensor has a low detection limit and good sensitivity, as shown in Table 1.

#### 4.5.1 Interference and selectivity

Difficulties in the accurate measurement of the concentration of l-Ala (Fig. S9A†) and l-Arg (Fig. S9B†) can arise from electroactive interfering amino acids such as histidine, lysine, glycine, methionine, l-asparagine (l-Asp), l-cysteine, and tyrosine, which are normally present in physiological samples. Thus, we tested the selectivity of the above biosensor design by adding the abovementioned seven interfering compounds at their typical concentrations (200 nM of the abovementioned interfering molecules). The introduction of CNTs deposited on the electrode surface dramatically reduced the sensitivity to the interfering compounds, while retaining the sensitivity for l-Ala and l-Arg. Furthermore, the compounds usually present in matrices where l-Ala and l-Arg are determined such as histidine, lysine, glycine, methionine, l-asparagine (l-Asp), l-cysteine, and tyrosine were tested for their ability as potential interferences, either directly electrochemically active at the potential used or indirectly as matrix components at the fixed potential of ±150 mV. Fig. S9† shows the amperogram recorded at the CoTANImMMPPc-CNTs/GCE composite biosensor under the experimental parameters employed for the detection of l-Ala and l-Arg. It can be observed that the newly developed biosensor did not exhibit any interference for the detection of the tested analytes, with negligible/minimal current responses for the interfering biomolecules, as shown in Fig. S9.†

### 4.6 Repeatability, reproducibility and stability of the CoTANImMMPPc/CNT electrode l-alanine and l-arginine sensors

The repeatability of the sensor was analyzed using the peak current values in the CV curves of the CoTANImMMPPc/CNT-GC electrode for the detection of 200 nM l-alanine and l-arginine in PBS (pH 7) for five successive measurements. The relative standard deviation (RSD) was less than 2.5%, indicating that there was no blocking effect on the oxidation products on the electrode surface. The fabrication reproducibility of six sensors, which were prepared using the same procedure, demonstrated acceptable reproducibility with an RSD of 1.9%. The stability of the l-alanine and l-arginine sensors was recorded using CV curves for 200 nM l-alanine and l-arginine in PBS (pH 7). It should be noted that the fabricated sensor was stored under ambient conditions. The peak current value was obtained at 7 day intervals. The results show that the current maintained about 95% of its initial value after 60 days, indicating the long-term stability of the sensor.

#### 4.6.1 Real sample analysis

Amperometric detection curves were obtained (inset in Fig. S10†) for the determination of l-arginine and l-alanine in peanuts and almonds (A) and egg and green beans (B) using CoTANImMMPPc/CNTs-GCE. During the amperometric detection, PBS (pH 7) electrolyte was added for each analysis. The current response observed was up to 95% within the 5–7 s after the sample was added and resultant amperogram was consistent with the lab sample results. The linearity was obtained from the concentration-dependent linear calibration plots, as shown in Fig. S10† ((A) peanuts and almonds and (B) eggs and green beans). The sensor parameters such as working range were calculated, and finally, it was found that it exhibited a more extensive linear range for the milk sample between 100 to 1800 nM (Fig. S10C and D†), and sensitivity and detection limit obtained for the seeds, eggs and vegetables were calculated. The linear range, LOD and sensitivity for l-arginine in the peanut and almond samples were determined to be 3 nM, 100 to 1800 nM, and 0.038 and 0.034 μA nM cm^−2^, respectively. For l-alanine in the egg and green bean samples, the LOD and sensitivity were 5 nM, and 0.032 and 0.029 μA nM cm^−2^, respectively. Thus, satisfying the requirements of a cheap and responsive electrochemical device, herein, an advantageous and instantaneous analytical device for the detection of l-arginine and l-alanine in peanut, almond, egg, and green bean samples was developed.

### 4.7 Electronic impedance spectroscopy

To study the nature of the electrode–electrolyte interface at the bare surface and modified electrodes, electrochemical impedance spectroscopy (EIS) is a suitable technique. The electron transfer resistance (*R*_ct_) at the electrode surface, which determines the electron transfer kinetics of the redox probe, can be calculated using the diameter of the semi-circle in the impedance spectrum.^47^ The present impedance spectra were compiled using an aqueous electrolyte solution of 0.1 M KCl. The obtained Nyquist plots for the CoTANImMMPPc/CNT-GC electrode (Fig. S11A†) and bare GCE (Fig. S11B†) show a significant difference in their response, as shown in Fig. S11A.† A semicircle with a larger diameter was observed for the CoTANImMMPPc/CNT-GC electrode in the frequency range of 100 kHz to 0.01 Hz. The charge transfer resistance (*R*_ct_) values obtained using the plots in Fig. S11† for the CoTANImMMPPc/MWCNT/GCE and bare GCE are 298 and 418, respectively.

## 5. Conclusion

Herein, a novel CoTANImMMPPc complex was synthesized and its structure was confirmed using various spectroscopic techniques. Also, an electrochemical investigation was performed using the CoTANImMMPPc/CNTs/GC electrode for the detection of l-alanine in the presence of l-arginine with the individual determination of two well-defined peaks by CV and DPV. These two amino acid analytes were detected at nanomolar concentrations, and the amperometry detection of the individual analytes and the selectivity of the electrode in the presence of other biomolecules such as l-cysteine, l-asparagine, ascorbic acid, dopamine, glucose and hydrogen peroxide were investigated, showing a negligible current response during the detection of l-alanine and l-arginine. The CoTANImMMPPc/MWCNT-GC electrode exhibits good analytical performances including low detection limit, repeatability, reproducibility, excellent linear dynamic range concentration range, and high selectivity and sensitivity.

## Conflicts of interest

The authors declare no conflicts of interest.

## Supplementary Material

RA-011-D1RA01815A-s001
