# Supplementary material for: Novel Schiff base cobalt(ii) phthalocyanine with appliance of MWCNTs on GCE: enhanced electrocatalytic activity behaviour of α-amino acids
Source: RSC Adv. 2021 May 6;11(27):16736–46. doi: 10.1039/d1ra01815a (PMC9032359; doi:10.1039/d1ra01815a)
Supplement: RA-011-D1RA01815A-s001 [file RA-011-D1RA01815A-s001.pdf]

Electronic Supplementary Material (ESI) for RSC Advances.
This journal is © The Royal Society of Chemistry 2021

Novel Schiff Base Cobalt (II) Phthalocyanine with Appliance of MWCNTs: Enhanced electrocatalytic activity behaviour of α-amino acids

Mounesh[1*], T. M. Sharanakumar[2], N. Y. Praveen Kumar[1], K. R. Venugopala Reddy[1], K. B. Chandrakala[1], L. Arunkumar[1], C. C. Vidyasagara[3]

[*1] Department of Studies and Research in Chemistry, Vijayanagara Srikrishnadevaraya University, Ballari-583105 Karnataka (India)

[2] Department of Studies and Research in Chemistry, Ballari Institute of Technology and Management, Ballari-583104 Karnataka (India)

[3] Department of Studies and Research in Chemistry, Rani Chennamma University, Belagavi-591156 Karnataka (India)

(Email: mounesh.m.nayak@gmail.com, Cell No: +91-8197546693)

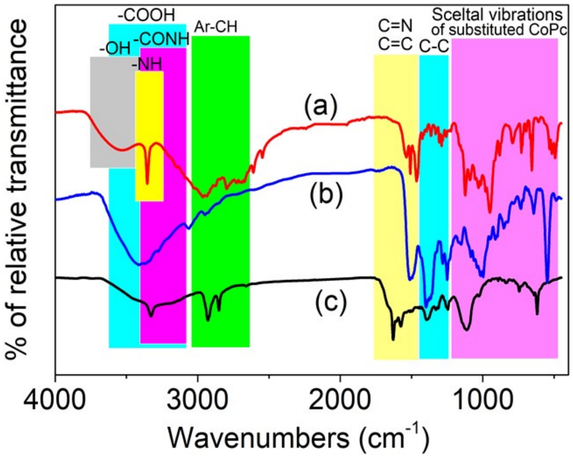

Fig.S1: FTIR spectra of (a) ANImMMP (b) CoTcPc and (c) CoTANImMMPPc

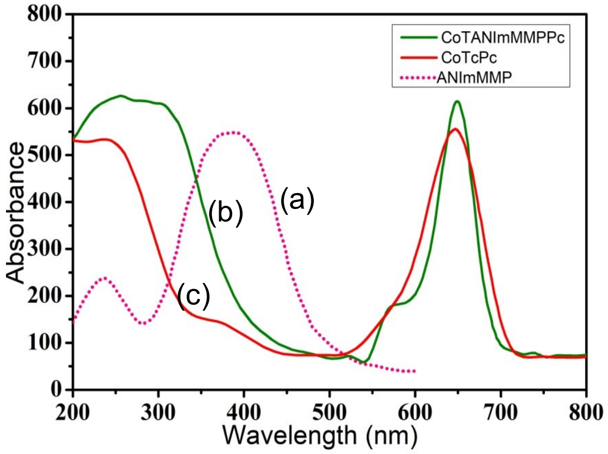

Fig.S2:UV-Visible spectra of (a) ANImMMP (b) CoTcPc and (c) CoTANImMMPPc

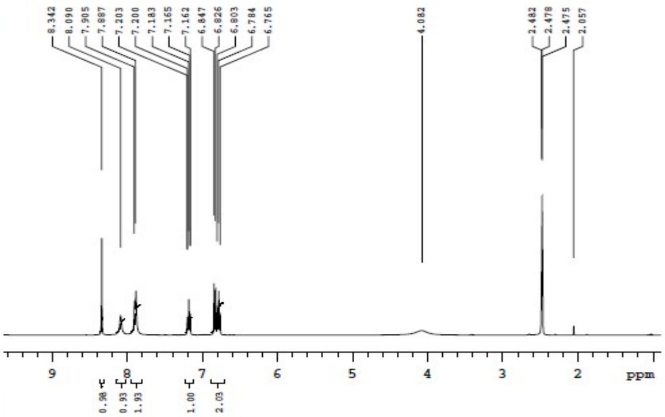

Fig.S3: ^{1}HNMR spectra of ANImMMP.

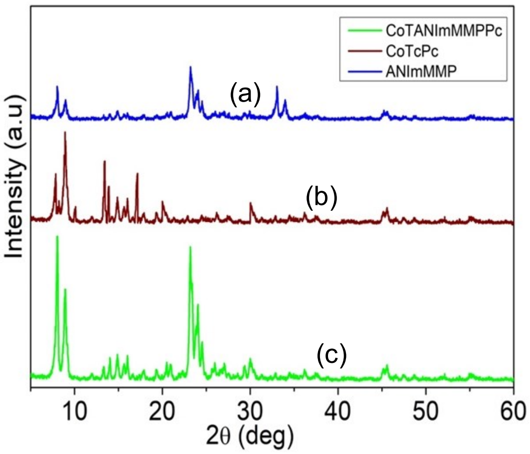

Fig.S4: XRD analysis of (a) ANImMMP, (b) CoTcPc and (c) CoTANImMMPPc.

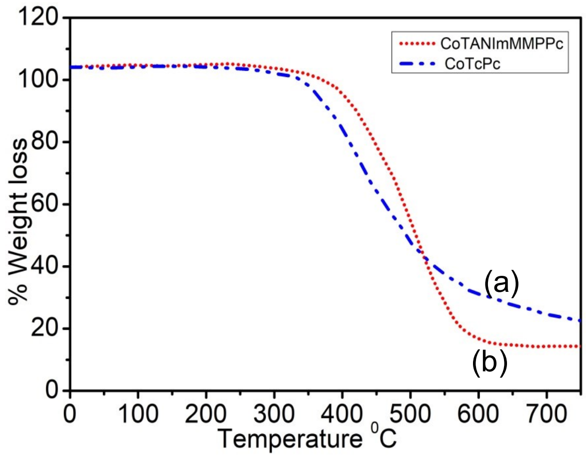

Fig.S5: TG analysis of (a) CoTCAPc and (b) CoTANImMMPPc.

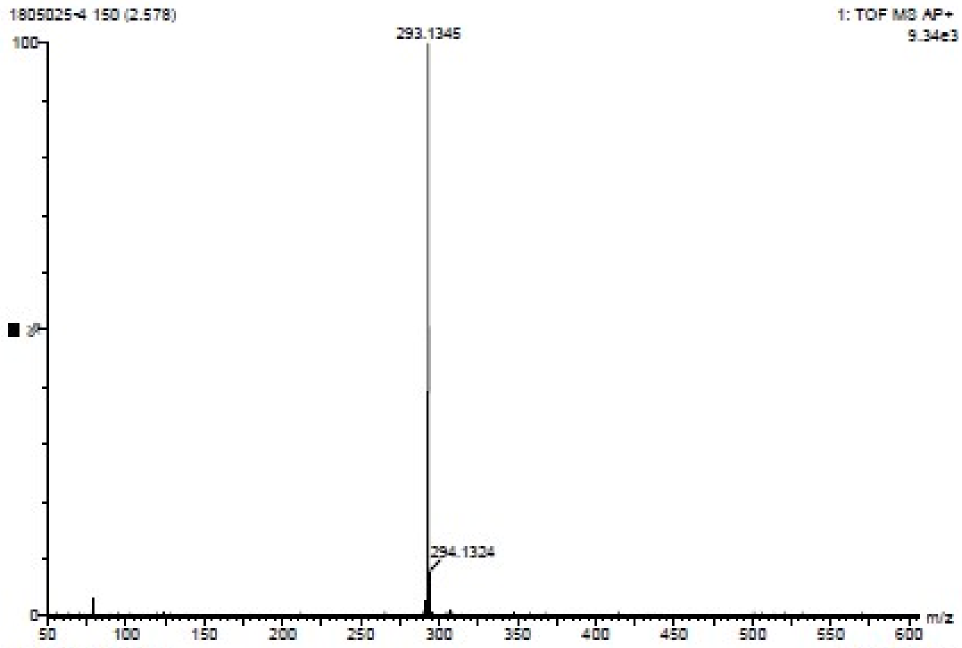

Fig.S6: Mass spectrum of ANImMMP.

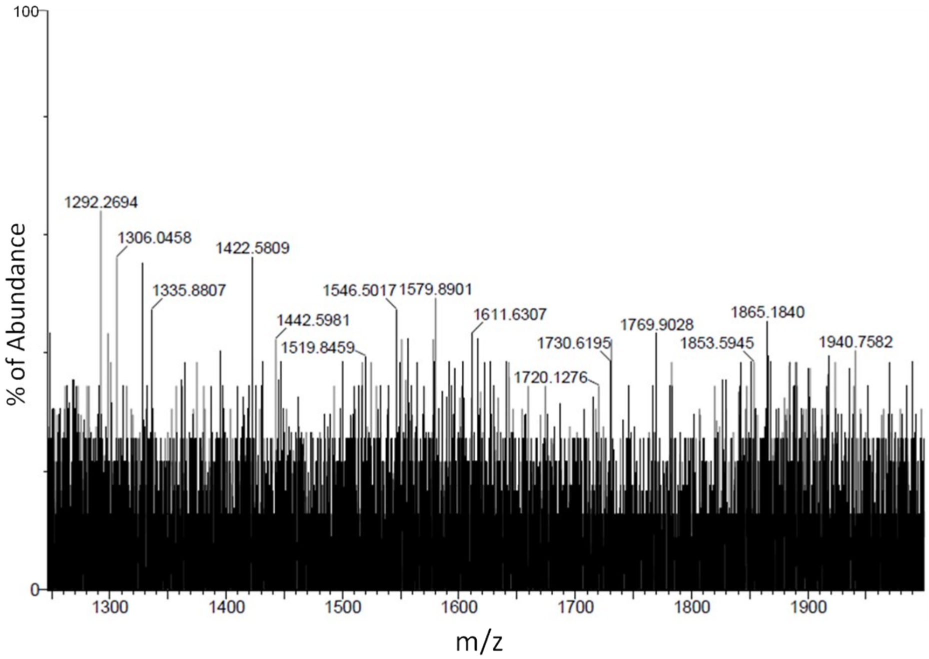

Fig.S7: ESI-MS mass spectrum of CoTANImMMPPc.

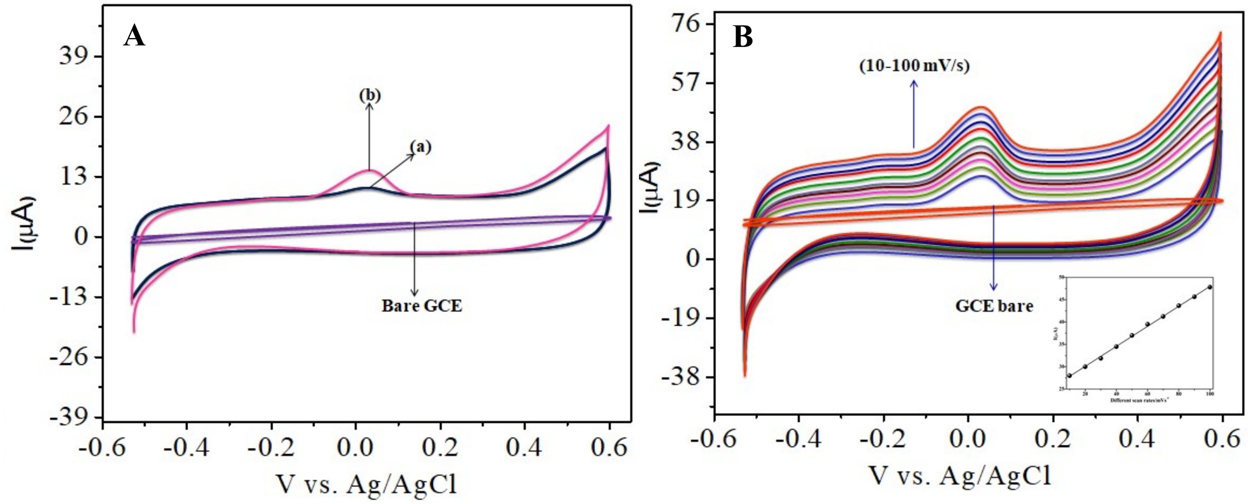

Fig.S8: Experimental CVs of modified GCE in (pH-7) PBS electrolyte solution at peaks: (A) inset bare GCE, (a) CoTANImMMPPc/GCE, (b) CoTANImMMPPc/CNTs/GCE; at scan rate 50 $mVs^{-1}$and (B) different scan rates of CoTANImMMPPc/CNTs/GCE/mVs^{-1}.

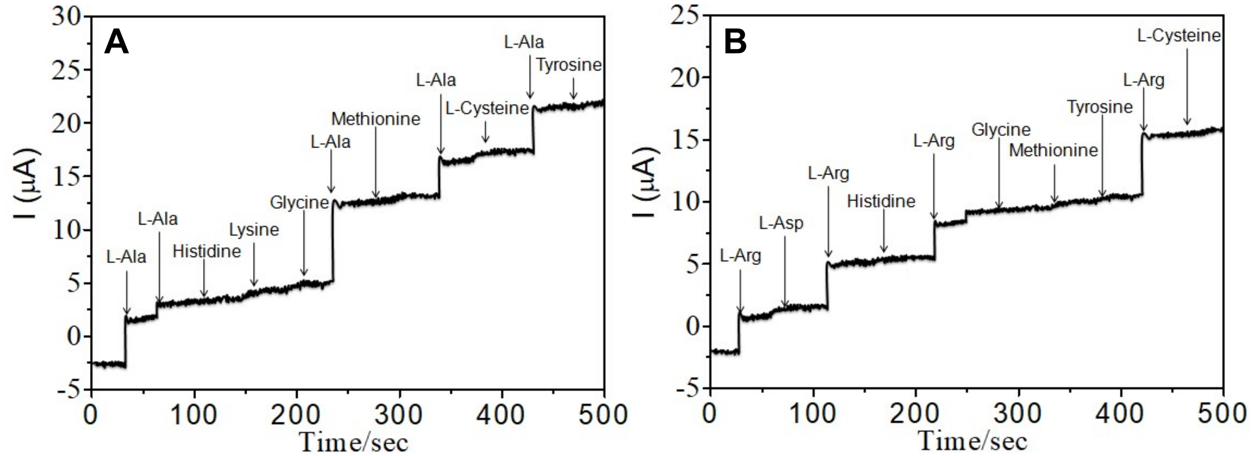

Fig.S9: Experimental amperometry interference responses of CoTANImMMPPc/CNTs-GCE in (pH-7) PBS at peaks: (A, B) 200 nM concentrations of Histidine, Lysine, Glycine, Methionine, L-Cysteine, Tyrosine, L-Asparginine by during L-Ala and L-Arg; applied potential ± 150 mV.

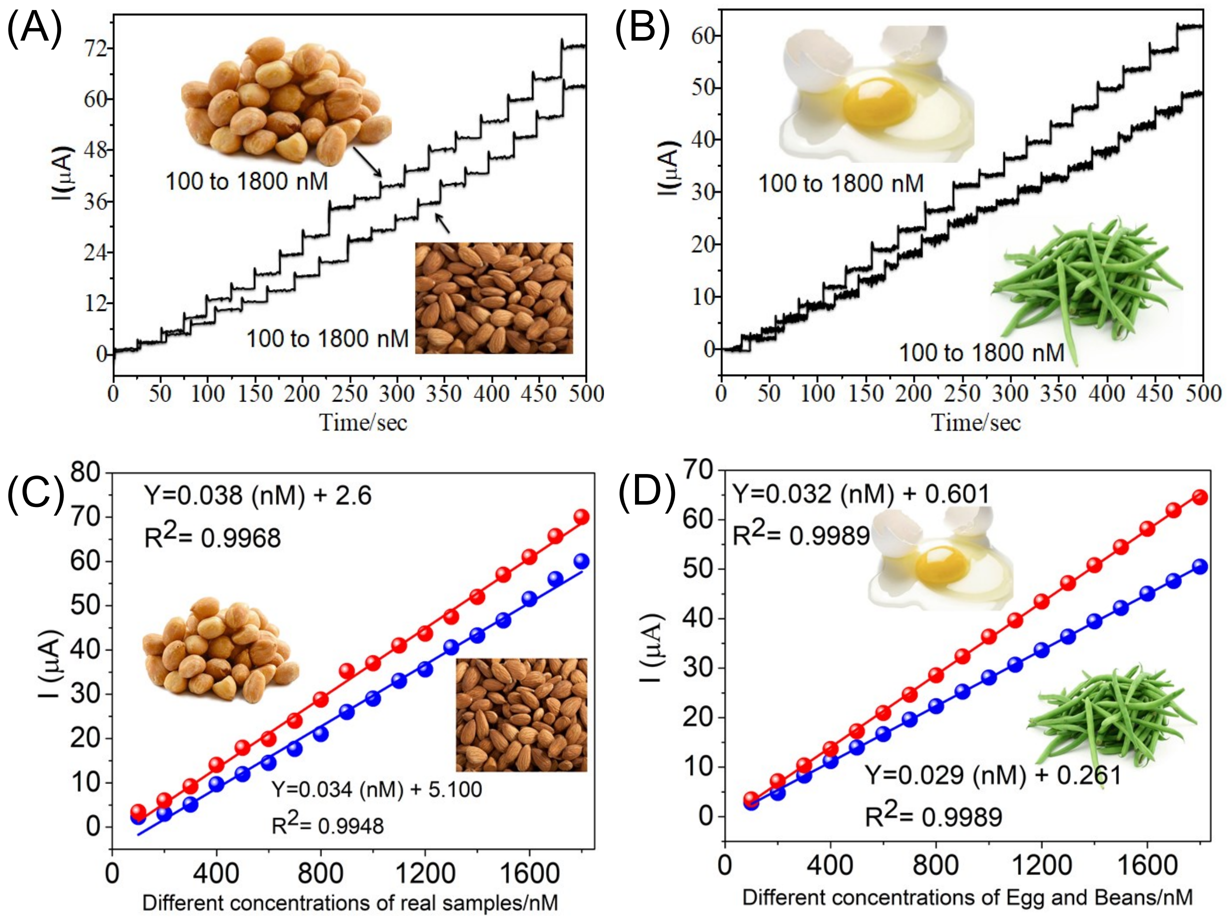

Fig.S10: Real sample analysis: Amperometric responses of CoTANImMMPPc/CNTs-GC electrode for each sequential addition of real samples containing L-Alanine and L-Arginine into continuously stirred phosphate buffer (pH-7). (A) Peanuts and Almonds, (B) Egg and Green Beans. Amperometric experiments are performed using CoTANImMMPPc/CNTs-GC electrode towards each sequential addition of real samples into phosphate buffer (pH-7). The rotation speed = 1500RPM and electrode potential = ± 150mV.

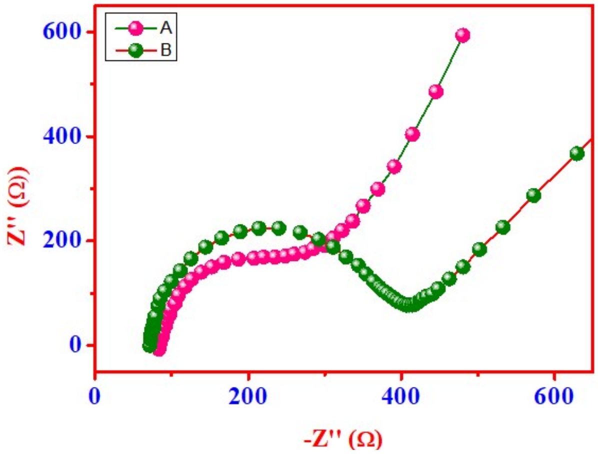

Fig.S11: Nyquist plot of A) CoTANImMMPPc/CNTs-GC and B) Bare GCE

Table S1: Elemental analysis of CoTANImMMPPc.

Compound Name (Yield) color	Empirical formula weight	Elemental analysis (%) Calcd: Found
CoTANImMMPPc (95%) Dark green	$C_{108}H_{79}CoN_{16}O_{12}$ (1851.52)	C; 70: (69.93)<br>H; 4.33: (4.27)<br>N; 12.14: (12.07)<br>O; 10.37: (10.25)<br>Co; 3.185: (3.15)
